# Perceptions of Social Support Sufficiency Among Older Adults With HIV in Sub-Saharan Africa

**DOI:** 10.1093/geroni/igab046.847

**Published:** 2021-12-17

**Authors:** Mark Brennan-Ing, Jennifer Kaufman, Kristen Porter, Catherine MacPhail, Janet Seeley, Stephen Karpiak

**Affiliations:** 1 Brookdale Center for Healthy Aging Hunter College, CUNY, New York, New York, United States; 2 John W. McCormack Graduate School of Policy and Global Studies, Moultonborough, New Hampshire, United States; 3 School of Health & Society, University of Wollongong, Bulli, New South Wales, Australia; 4 London School of Hygiene and Tropical Medicine, London, England, United Kingdom; 5 GMHC, National Resource Center on Aging and HIV, New York, New York, United States

## Abstract

Globally, the greatest number of older people with HIV (OPWH) are in sub-Saharan Africa (3.7 million). This population will continue to expand with greater access to anti-retroviral therapy. Compared to OPWH in high income counties, these OPWH have constrained access to government and community-based services and largely rely on assistance from family, friends, and neighbors for their social support needs. We examined factors related to perceptions of instrumental and emotional support sufficiency (availability and adequacy) among OPWH age 50 and older in Uganda (n = 101) and South Africa (n = 108). Significant covariates of instrumental support sufficiency included not having an AIDS diagnosis, greater support from family, and less support from friends. Significant covariates of emotional support sufficiency were fewer depressive symptoms, greater support from family, and geographic location (Uganda). Explanation of these findings based on social network characteristics and implications for policy and program development will be discussed.

